# Oncolysis of malignant human melanoma tumors by Coxsackieviruses A13, A15 and A18

**DOI:** 10.1186/1743-422X-8-22

**Published:** 2011-01-18

**Authors:** Gough G Au, Leone G Beagley, Erin S Haley, Richard D Barry, Darren R Shafren

**Affiliations:** 1 The Picornaviral Research Unit, The School of Biomedical Sciences and Pharmacy, Faculty of Health, The University of Newcastle, Newcastle, New South Wales, 2300, Australia; 2 Newcastle Innovation Ltd, Industry Development Centre, University Drv, Callaghan, New South Wales, 2308, Australia; 3 Viralytics Ltd, Suite 1B, 55-63 Grandview St, Pymble, New South Wales, 2073, Australia

## Abstract

Many RNA viruses are displaying great promise in the field of oncolytic virotherapy. Previously, we reported that the picornavirus Coxsackievirus A21 (CVA21) possessed potent oncolytic activity against cultured malignant melanoma cells and melanoma xenografts in mice. In the present study, we demonstrate that three additional Group A Coxsackieviruses; Coxsackievirus A13 (CVA13), Coxsackievirus A15 (CVA15) and Coxsackievirus A18 (CVA18), also have similar oncolytic activity against malignant melanoma. Each of the viruses grew quickly to high titers in cancer cells expressing ICAM-1 and intratumoral injection of preformed subcutaneous SK-Mel-28 xenografts in mice with CVA13, CVA15 and CVA18 resulted in significant tumor volume reduction.

As preexisting immunity could potentially hinder oncolytic virotherapy, sera from stage IV melanoma patients and normal controls were tested for levels of protective antibody against the panel of oncolytic Coxsackieviruses. Serum neutralization assays revealed that 3 of 21 subjects possessed low levels of anti-CVA21 antibodies, while protective antibodies for CVA13, CVA15 and CVA18 were not detected in any sample. Serum from individuals who were seropositive for CVA21 failed to exhibit cross-neutralization of CVA13, CVA15 and CVA18. From these studies it can be concluded that the administration of CVA13, CVA15 or CVA18 could be employed as a potential multivalent oncolytic therapy against malignant melanoma.

## Findings

Numerous viruses from a diverse range of virus families are being identified for use as oncolytic virotherapy agents. The underlying principle of oncolytic virotherapy is that the specificity of lytic viral infection can be harnessed to destroy malignant cells selectively, whilst leaving normal host cells intact. Previously we have shown that Coxsackievirus A21 (CVA21) can selectively infect and destroy *in vitro *cultures of malignant melanoma cells that characteristically over-express intercellular adhesion molecule-1 (ICAM-1) and/or decay accelerating factor (DAF) [1,2]. The genetically unmodified prototype CVA21 (Kuykendall strain) is also effective *in vivo*, eliminating tumor burden in NOD-SCID mice bearing subcutaneous melanoma xenografts following a single injection of virus [2].

Malignant melanoma is a cancer of the pigment producing cells of the skin (melanocytes), and arises from the uncontrolled proliferation of these cells. Once the cancer has metastasized, it is largely incurable, despite surgery or treatment with intensive cycles of chemotherapy or radiation therapy [3]. In an attempt to distinguish the cell adhesion molecules involved in tumor progression and metastasis, researchers have identified the cell surface molecule ICAM-1, as a progression marker for metastatic melanoma [4-7]. Concurrently, ICAM-1 is also recognized as an attachment receptor for many enteroviruses including CVA13, CVA15, CVA18 and CVA21 [8,9].

Functionally, the expression of the ICAM-1 receptor in normal tissue allows for i) cellular contact between neighboring cells, ii) signaling in inflammatory processes and iii) the activation of the T-cell mediated host defense system [10]. It is hypothesized that the over-expression of ICAM-1 on melanoma cells may have a role in the interference of normal immune function [10], as well as assisting melanoma metastasis through cellular interactions with circulating lymphocytes via the surface expressed lymphocyte function-associated antigen-1 (LFA-1) integrin molecule [4,7,11,12].

As CVA21 is a naturally occurring virus that circulates occasionally in the community, one concern regarding its use as an anti-cancer therapy is the presence of pre-existing immunity in the recipient cancer patient. Information concerning the epidemiology and prevalence of CVA21 infection in the community is scanty, but a 1959 study in Great Britain found that 36.1% of males and 18.4% of females (inclusive of all age groups), possessed serum antibodies to a virus identical to the Coe strain of CVA21 [13]. The Coe strain was first isolated from throat swabs of military recruits suffering from mild acute respiratory illnesses in California [14], and is serologically similar to the Kuykendall strain [13]. A more recent study of enterovirus infections in Scottish blood donors failed to detect amplifiable CVA21 template from a total of 3658 pools of 95 donations tested, however these samples were not tested for neutralizing antibody status [15].

A potential strategy for successful ongoing ICAM-1 targeted virotherapy that delays or avoids the impact of virus neutralization, is to use a subset of Coxsackieviruses that are serologically unrelated but that all recognize the same cellular-uptake receptors. The three Coxsackie A group viruses, CVA13, CVA15 and CVA18, were previously shown to employ ICAM-1 for binding and cell infectivity [8]. Based on these findings, CVA13, CVA15 and CVA18 were evaluated as potential candidates for the ICAM-1 targeted oncolytic therapy of malignant melanoma.

Coxsackievirus A13, CVA15, CVA18 and CVA21 belong to the C cluster of human enteroviruses (HEV-C), and are members of the *Picornaviridae *family [16]. Picornaviruses are small icosahedral, non-enveloped viruses that contain a single strand of positive sense RNA [17]. Clinically, the majority of human enterovirus infections are asymptomatic [17]. While the Kuykendall strain of CVA21 is frequently associated with the "common-cold", it is important to note that the prototype strains of CVA13 (Flores), CVA15 (G-9) and CVA18 (G-13) were originally isolated from stool samples of patients displaying no detectable illness [18].

In this study the oncolytic potential of CVA13, CVA15 and CVA18 was established by using both *in vitro *melanoma cultures, and *in vivo *melanoma xenografts. The frequency of CVA13, CVA15 and CVA18 infections in the community or the prevalence of specific-neutralizing antibody levels against these viruses in individuals is difficult to predict due to a lack of sero-epidemiological studies. We examined sera from melanoma patients with terminal disease (*n *= 15) as well as sera from healthy volunteers (*n = *6) for virus specific-neutralizing antibody levels.

## Lytic activity of CVA13, CVA15, and CVA18 in *in vitro *melanoma cell culture

**Figure 1:**
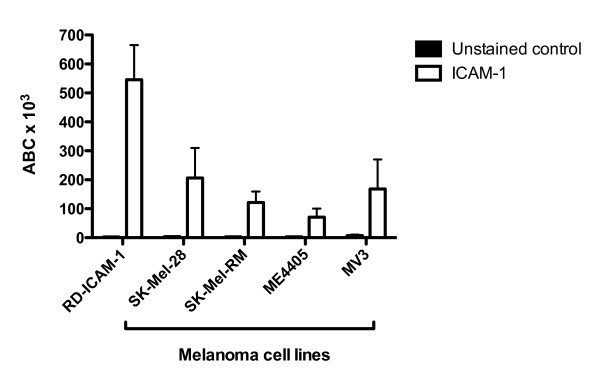
**ICAM-1 expression on SK-Mel-28, ME4405 and RD-ICAM-1 cells**. Quantitative flow cytometric analysis of virus-entry receptor ICAM-1 expression on melanoma cell lines SK-Mel-28, SK-Mel-RM, ME4405, MV3; and ICAM-1 transfected RD cell line (RD-ICAM-1). The level of ICAM-1 antibodies bound per cell (ABC) are denoted by the white bars whereas the unstained control is shown in black.

As a first approach, surface levels of ICAM-1 were examined on the melanoma cell lines SK-Mel-28, SK-Mel-RM, ME4405 and MV3. RD-ICAM-1 cells were used as a positive control. This cell line was produced by the stable transfection of rhabdomyosarcoma cells (RD) with cDNA encoding the ICAM-1 molecule and is used as a reference cell line in our laboratory [9]. These cells were maintained in DMEM containing 10% FCS and harvested using versene prior to flow cytometry. Commercially available PE-conjugated antibodies against ICAM-1 (ab1822) were obtained from Abcam. To assess the surface expression of ICAM-1 on melanoma cells, these antibodies were used together with QuantiBRITE™ PE beads (Becton Dickinson) according to manufacturer's instructions to determine the approximate number of antibodies bound per cell (ABC). Flow cytometric analysis confirmed the presence of surface ICAM-1 expression on the four melanoma cell lines SK-Mel-28, SK-Mel-RM, ME4405 and MV3; and also on the ICAM-1 transfected RD cells (Figure 1).

**Figure 2:**
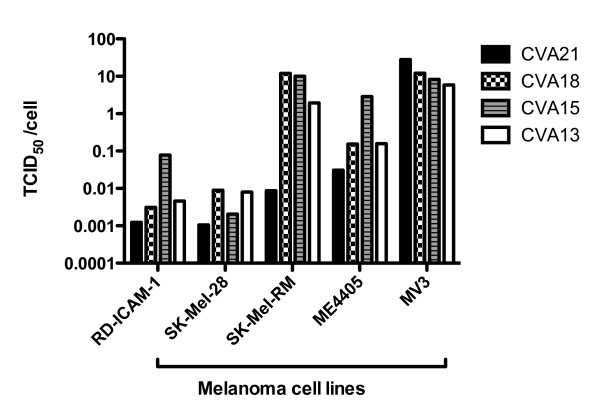
**Destruction of melanoma cells by CVA13, CVA15, CVA18 and CVA21**. Cultures of SK-Mel-28, SK-Mel-RM, ME4405, MV3 and RD-ICAM-1 monolayers were infected with 10-fold serial dilutions of virus ranging from 1:10 to 1:10^7^. After incubation for 48 h, the plates were fixed and stained with a crystal violet/methanol and TCID_50_/cell required to induce monolayer destruction was calculated. SK-Mel-28 and RD-ICAM-1 were the most sensitive lines to the panel of CVAs requiring only low concentrations of virus to achieve cell lysis.

As CVA13, CVA15, and CVA18 utilize ICAM-1 for virus-cell entry, these viruses were evaluated for their oncolytic activity against the panel of melanoma cells and the control cell line RD-ICAM-1. Prototype strains of Coxsackie A viruses (CVAs), CVA13 (Flores), CVA15 (G-9), CVA18 (G-13), CVA21 (Kuykendall) were obtained from Dr M. Kennett. Confluent monolayers of SK-Mel-28, ME4405, MV3, SK-Mel-RM and RD-ICAM-1 cells in 96-well tissue culture plates were inoculated with 10-fold serial viral dilutions (100 μl/well in quadruplicate) of either CVA21, CVA18, CVA15 or CVA13 and incubated at 37 °C in a 5% CO_2 _environment for 48 h. Cytopathic effects (CPE) at each dilution was examined by microscopy and fifty percent viral endpoint titers calculated using the Karber method [19]. CVA21 exhibited potent lytic activity, such that monolayers of SK-Mel-28 cells were destroyed even at a level of approximately 0.001 TCID_50_/cell (Figure 2). The subset of viruses, CVA13, CVA15 and CVA18 displayed comparable levels of oncolytic activity on SK-Mel-28 monolayers. SK-Mel-RM, ME4405 and MV3 cells when infected with the panel of CVAs also demonstrated rapid cell death, however higher concentrations of virus were required compared to the RD-ICAM-1 and SK-Mel-28 cell lines.

## CVA13, CVA15 and CVA18 exhibit similar replication profiles in RD-ICAM-1 cells and melanoma cell lines SK-Mel-28 and ME4405

The viral replication rate and release of progeny virus from infected tumor cells are important factors for successful oncolytic virotherapy. We sought to determine the growth properties of the Coxsackie A virus subset (CVA13, CVA15 and CVA18) in SK-Mel-28, ME4405 and RD-ICAM-1 cells compared to human PBMCs. Human peripheral blood mononuclear cells (PBMCs) were isolated from healthy adults by Ficoll-Paque gradients and cultured in RPMI media with 10% FCS. For the adherent cell lines, confluent cell monolayers in 6-well plates were infected with approximately 10^6 ^TCID_50 _of CVA13, CVA15 or CVA18 (multiplicity of infection (MOI) ~ 1 TCID_50_/cell). The plates were incubated for 1 hr at 37°C, then washed 3 times each with DMEM and the monolayers overlaid with 3 ml of DMEM containing 2% FCS before incubating at 37°C. Synchronized infection was interrupted at time intervals of 0, 2, 4, 6, 8, 10, 12, 24 and 48 hours. The cell monolayers were lysed by three consecutive freeze-thaw cycles before the viral yield in the cell lysate was determined in an endpoint titration assay.

The growth of the three CVAs in PBMCs was carried out in suspension and these cells were infected at a MOI of 1 TCID_50_/cell with either CVA13, CVA15 or CVA18. Virus was allowed to bind for 1 hour prior to washing three times with RPMI media (2% FCS). For each virus, the infected PBMCs were then divided into four tubes with approximately 8.5 × 10^5 ^cells per tube in a final volume of 500 μl of RPMI (2% FCS). These infected cells were then incubated at 37°C and total cell suspensions at 0, 12, 24 and 48 hours were lysed by three consecutive freeze-thaw cycles before being titrated to determine virus yield.

**Figure 3:**
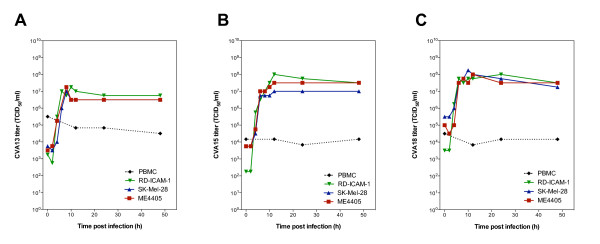
**Growth curves of CVA13, CVA15 and CVA18 in SK-Mel-28, ME4405 and RD-ICAM-1 cells compared to PBMCs**. SK-Mel-28, ME4405, RD-ICAM-1 and PBMCs were challenged with CVA13 (A), CVA15 (B) or CVA18 (C) for 1 h, and after the removal of unbound virus, cells and media were collected at the indicated time points for assessment of virus progeny by endpoint titration.

The growth curves revealed that CVA13, CVA15 and CVA18 underwent exponential replication between 4 and 6 hours, and reached maximal titers in SK-Mel-28, ME4405 and RD-ICAM-1 within 12 hours post infection (Figure 3A,B and 3C). Irrespective of the different tumor cell lines, CVA13, CVA15 and CVA18 displayed similar replication rates and yielded increases in virus output by approximately 10^4 ^fold. As CVA21 spreads via the systemic circulation after virus therapy of *in vivo *melanoma tumors [2], we investigated the likelihood that human PBMCs could be exposed to viral challenge. Normal PBMCs however, were refractile to viral infection, exhibiting no exponential increase in CVA13, CVA15 or CVA18 titers compared to those seen in the SK-Mel-28, ME4405 or RD-ICAM-1 cell lines (Figure 3). Flow cytometric analysis revealed low levels of ICAM-1 on the surface of PBMCs and upon further incubation with virus for 6 days, no increase in virus yields were detected (data not shown).

## CVA13, CVA15 and CVA18 effectively reduce tumor volumes of subcutaneous SK-Mel-28 xenografts in a SCID mouse model

To evaluate the potential use of these viruses for the control of melanoma tumor progression *in vivo*, preformed SK-Mel-28 tumors (~ 150 mm^3^) on the hind flank of SCID mice, were directly injected with a single dose of either PBS, live CVA13, CVA15 or CVA18 (~ 10^5 ^TCID_50_). All animal work was performed under guidelines approved by The University of Newcastle Animal Care and Ethics Committee. SK-Mel-28 cells grown in DMEM containing 10% FCS, were harvested, washed twice with DMEM, and resuspended in sterile PBS. Tumor cells were xenografted into the flanks of anaesthetized 4-6 week old female SCID mice by single subcutaneous injections of 1 × 10^6 ^cells (*n *= 20). Once solid palpable tumors were established, the animals were divided into four treatment groups, each animal receiving either intratumoral injections of PBS (0.1 ml), CVA18, CVA15 or CVA13 (0.1 ml containing approximately 10^5 ^TCID_50 _units). Melanoma xenograft growth was monitored weekly with calipers and averages of tumor volumes (based on the volume of a spheroid) from each treatment group were plotted ± standard error.

**Figure 4:**
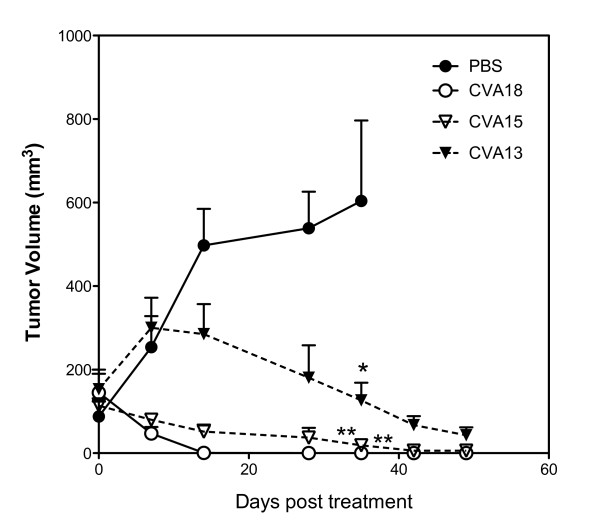
**SK-Mel-28 tumors are responsive to intratumoral CVA13, CVA15 and CVA18 therapy in SCID mice**. Severe combined immunodeficient (SCID) mice bearing preformed s.c. tumors (approximately 150 mm^3^) growing on the flanks after injection of 1 × 10^6 ^SK-Mel-28 cells, received an intratumoral injection with a single dose of CVA13, CVA15, CVA18 (10^5 ^TCID_50_) or PBS (n = 5 for each group). The average relative tumor sizes were measured externally with calipers and are expressed as the means of five treated mice ± S.E. * and ** indicate statistical significance of tumor volumes compared to the control, *P *< 0.05 and *P *< 0.01 respectively (One-way ANOVA).

Significant reductions in tumor volumes were observed 35 days post CVA13, CVA15 and CVA18 treatment compared to the PBS treated control group (One-way ANOVA, *P *< 0.01 for both CVA18 and CVA15 vs. PBS, *P *< 0.05 for CVA13 vs. PBS group) (Figure 4). The groups of animals receiving CVA13, CVA15 and CVA18 intratumoral therapy failed to display any further increases in tumor volumes even up to 48 days post treatment. In contrast the tumors of PBS treated mice steadily increased, with animals from this group being euthanized at 35 days post treatment due to ethical considerations. CVA18 appeared to be rapidly effective against preformed SK-Mel-28 tumors, with aggressive tumor reduction leading to the complete clearance of tumors (5/5 mice tumor free), compared to CVA15 (2/5 mice tumor free) and CVA13 (0/5 tumor free) at day 48 post treatment. CVA13 was much slower to induce anti-tumor activity against the preformed SK-Mel-28 tumors and failed to induce complete tumor regression despite showing initial signs of tumor reduction.

## Levels of serum neutralizing antibodies to CVA13, CVA15, CVA18 and CVA21 in melanoma patient sera

To gauge the extent to which melanoma patients may have been naturally exposed to CVA21, CVA13, CVA15 and CVA18, fifteen late stage melanoma patients and six healthy volunteers were tested for the presence of virus specific neutralizing antibodies. Heat inactivated serum samples were diluted in DMEM (2% FCS) 1:4 to 1:256. One hundred microliters of each serum dilution was incubated with 100 μL of each respective virus (100 TCID_50 _of either CVA21, CVA18, CVA15 or CVA13) at 37 °C for 1 hour. Two hundred microliters of this serum/virus mixture was then plated in triplicate on SK-Mel-28 cells. As a control, two commercially available stocks of pooled gamma globulin (Commonwealth Serum Laboratories, Victoria, Australia) were tested for neutralizing antibodies against CVA13, CVA15, CVA18 and CVA21 as per the above protocol. Plates were incubated for 3 days, then examined microscopically for the development of CPE, and then stained with crystal violet/formalin. The neutralization titers were calculated using the Karber method [19], with serum neutralizing titers > 1:4 considered to be positive.

Pre-existing neutralizing antibodies against CVA13, CVA15 or CVA18 were not detected in the serum of the melanoma patients, healthy volunteers, or the commercial pooled-IgG preparation (Table 1). Three of the fifteen melanoma patients demonstrated circulating CVA21 antibody titers >1:4, while no detectable levels of CVA21 antibodies were found in the serum of the healthy control group. Serum from patients who were positive for CVA21 neutralizing antibodies, failed to offer cross-protective neutralization against CVA13, CVA15 or CVA18. The two samples of commercial pooled-immunoglobulin IgG, neutralized 100 TCID_50 _of CVA21 but not CVA13, CVA15 or CVA18 (Table 1). As the normal pooled immunoglobulin is prepared from a diverse population of healthy blood donors, these data support the postulate that natural CVA13, CVA15 and CVA18 infections are uncommon in the community.

**Table 1:** Serum neutralization titers to CVA13, CVA15 and CVA18.

Sample group	No. sera	No. CVA13-positive (%)	No. CVA15-positive (%)	No. CVA18-positive (%)	No. CVA21-positive (%)
Commercial immune globulin	2	0 (0)	0 (0)	0 (0)	2 (100)
					
Melanoma	15	0 (0)	0 (0)	0 (0)	3 (20.0)
Control	6	0 (0)	0 (0)	0 (0)	0 (0.0)
**Total patients**	**21**	**0**	**0**	**0**	**3 (14.3)**

Fifteen melanoma patients were examined for serum neutralization of CVA21, CVA13, CVA15 and CVA18. Heat inactivated serum samples were diluted serially, and then titrated against 100 TCID_50 _(CVA21, CVA13, CVA15 and CVA18) to assess neutralization status. Fifty percent neutralization scores of 1:4 or above calculated by the Karber method [19], were considered to be positive for neutralization status. Six healthy volunteers were also assessed for serum neutralizing antibodies against CVA13, CVA15 and CVA18, all of which were below 1:4 (50% neutralization score). Two commercially available immune serum globulin samples were also tested for neutralizing capacity.

## Conclusions

Coxsackieviruses A13, CVA15 and CVA18 are an enteroviral subset that potentially may be used to destroy malignant melanoma tumor cells because their entry receptor ICAM-1 is abundantly expressed on the surface of melanoma cells. *In vitro *and *in vivo *findings in the present study confirms the successful targeting of human melanoma cells and induction of tumor lysis by CVA13, CVA15 and CVA18.

The role of the immune system in preventing the systemic traffic of oncolytic viruses is a potential impediment to the development of effective virotherapy strategies. The presence of specific neutralizing antibodies that prevent virus attachment and infection may therefore limit successful virotherapy. An important outcome of this study was that neutralizing antibodies to CVA13, CVA15 and CVA18 were not detected in any of the melanoma patient samples tested, and those that were seropositive to CVA21, failed to cross-protect against CVA13, CVA15 and CVA18 challenge.

Our findings suggest that the mildly pathogenic viruses CVA13, CVA15 and CVA18, are potent oncolytic agents and viable candidates for a sequential multivalent viral oncolytic therapy. As a treatment strategy, sequential infection by these similar but serologically distinct CVAs may be a realistic means of circumventing the impact of anti-viral neutralizing antibodies.

## Abbreviations

CPE: Cytopathic Effect; CVA13: Coxsackievirus A13; CVA15: Coxsackievirus A15; CVA18: Coxsackievirus A18; CVA21: Coxsackievirus A21; DMEM: Dulbeccco Modified Eagle's Medium; FCS: Fetal Calf Serum; MOI: Multiplicity of Infection; PBMCs: peripheral blood mononuclear cells; RPMI: Roswell Park Memorial Institute.

## Competing interests

Darren R. Shafren is a director of Viralytics Ltd.

Richard D. Barry and Darren R. Shafren declare a financial interest in Viralytics Ltd.

## Authors' contributions

DRS and GGA designed the research. GGA performed the experimental work, conducted the data analysis and drafted the manuscript. LGB and ESH contributed to the testing of patient samples. All participated in the review of the manuscript. All authors read and approved the final manuscript.
